# Some Effects of Adenoids

**Published:** 1907-12-21

**Authors:** 


### Laryngology and Rhinology.

### SOME EFFECTS OF ADENOIDS.

The effects of adenoid growths on the general health, the shape of the thorax, the condition of the lungs, or in producing diseases of the ears have often been described; but it is also interesting to consider how nasal obstruction in childhood alters the shape of the face, nose, and mouth, and causes persistent obstruction after removal of the cause. It is well known that adenoid growths tend to contract about the age of puberty, and even now one occasionally hears how the doctor has said that the child will "grow out of it." It is true that, when one encounters a small pad of adenoids in a patient approaching puberty, the slight symptoms of which can be kept in abeyance by the use of a simple nasal lotion, one may safely await the time of their atrophy and of the expansion of the naso-pharynx. But too often the adenoids, before their disappearance, have already so modified the growth and shape of the nose and mouth that mouth-breathing becomes permanent, and that in a form most difficult to treat. A shrivelled pad of adenoids affords a lodgment for decomposing secretions, and becomes a cause of troublesome post-nasal catarrh; but even more important are the results of long-continued nasal obstruction brought about in a simple mechanical manner. At first sight it may appear an exaggeration to ascribe such considerable changes to so slight a cause, until it be remembered how powerful an action a slight force may have if it be exerted over a prolonged period on tissues in the plastic stage of growth.

At birth the jaws are disproportionately small; the antrum is represented by a mere cleft, and the germs of the permanent molar teeth lie high up above the level of the alveolar arch. At the period of the second dentition the antrum expands rapidly, the tuberosity of the superior maxilla is developed, and the permanent molars are forced downwards into their final position. In infancy, too, the ramus of the lower jaw forms a very obtuse angle with the body, but adaptation to the altered shape of the superior maxilla now causes it to approach a right angle, as a result of the steady pressure exerted by the upper molar teeth upon those of the lower jaw.

Now, when the mouth is kept shut, the tongue exerts an outward pressure upon the molar teeth and the sides of the alveolar arch, and at the same time a certain amount of suction, or negative pressure, is produced between the roof of the palate and the dorsum of the tongue which helps to sustain the weight of the lower jaw. In this way the dental arch is widened, and becomes well rounded in front, while the arch of the palate is rendered wide and flat; this has the effect of making the nasal fossæ both wider and deeper. When, however, the mouth is habitually open these forces all become inoperative, and other forces, which act in the opposite direction, are produced. The lower jaw drops, and is partly supported by the tissues of the cheeks, which thus exert an inward pressure upon the molar teeth. The cheeks are pulled downwards, and with them the alæ nasi, so that the nostrils assume a narrow slit-like form; the dilatator muscles of the alæ are little used and do not develop, the alæ therefore collapse like valves on inspiration. This condition of "alar collapse" is the most frequent and intractable cause of disappointment after operations for neglected and long-continued nasal obstruction. The lower jaw is seldom in contact with the upper, and does not become moulded to the usual adult shape; the angle remains obtuse, so that only the molar teeth come into contact, while in front the incisors do not meet. In the upper jaw the dental arch is narrow and pointed in front, the incisor teeth are prominent and crowded together, and project in front of those of the lower jaw. The bony palate is narrow and very high, forming a steep vault like a Gothic arch; the nasal passages are thus encroached upon and are unduly narrow. Is it surprising that, when all this has been allowed to occur, the removal of the adenoids fails to relieve nasal obstruction? It is, therefore, most important that children who have enough adenoid hypertrophy to produce definite nasal obstruction should receive special attention at the age of six to eight years, when development of the jaws is most active, and that the adenoids should be removed before irremediable deformity is produced. Dentists who have to treat crowding of the front teeth should always inquire carefully about nasal obstruction, for this is the almost constant cause of this deformity.

In addition to these effects, a marked degree of adenoid hypertrophy is always associated with chronic rhinitis which, if long continued, produces hypertrophy of the turbinate bodies. In young children this will generally disappear after the removal of the adenoids, but in cases over the age of ten or twelve it is wise to remove the posterior ends of the inferior turbinals at the time of the operation, for the hypertrophy is usually most marked behind. This adds little to the severity of the operation, and is easily done by passing a snare along the nose, meeting it with the index finger introduced through the mouth, and hooking the loop around the part to be removed.

In cases with alar collapse and ill-developed nostrils it must not be supposed that simple removal of the obstruction will at once effect a complete cure. The patient must be accustomed to use the nose, and the alæ nasi must be expanded by appropriate exercises. In slight cases these are of great value without operation; but when there is marked obstruction respiratory exercises are most fatiguing, and do little or no good. The simplest and most effective exercise is as follows: The patient stands erect and slowly elevates the arms with the elbows straight until the hands meet above the head, taking a deep inspiration the while through the nose, with the mouth firmly closed; he then slowly, during expiration through the nose, depresses the arms until they hang by the side. This is repeated eight or ten times a minute. The exercise must not be continued long enough to tire the patient; twenty times twice a day is sufficient for weakly people, or for the beginning of the treatment, and the frequency may be gradually increased.

